# Development and Validation of a Machine Learning Model to Predict Postoperative Morbidity and Mortality in Patients With COVID-19 Who Underwent Major Surgery

**DOI:** 10.7759/cureus.91247

**Published:** 2025-08-29

**Authors:** Sean P McDermott, Leming Zhou, Ata Murat Kaynar

**Affiliations:** 1 Department of Anesthesiology and Perioperative Medicine, University of Pittsburgh Medical Center, Pittsburgh, USA; 2 Department of Health Information Management, School of Health and Rehabilitation Sciences, University of Pittsburgh, Pittsburgh, USA; 3 Department of Critical Care Medicine, University of Pittsburgh Medical Center, Pittsburgh, USA

**Keywords:** ai and machine learning, covid-19, national surgical quality improvement program (nsqip), postoperative outcome, predictive analytics

## Abstract

Active coronavirus disease (COVID-19) increases the risk of postoperative morbidity and mortality. This study applied machine learning (ML) algorithms to predict postoperative outcomes using preoperative features. Multiple supervised ML models were trained on 153 features from 10,613 patients with COVID-19 who underwent major surgery in 2022 and validated on 5,269 patients from 2021. Among patients with COVID-19, 906 (17.2%) in 2021 and 1,248 (11.8%) in 2022 experienced significantly higher 30-day composite mortality or major morbidity compared with 31,019 (3.2%) of 978,582 patients without COVID-19 in 2021 and 29,874 (3.0%) of 1,001,286 patients without COVID-19 in 2022 (p < 0.001). LightGBM was the best-performing algorithm, achieving an area under the receiver operating characteristic curve (AUC) and F1 score of 0.865 and 0.512 when trained on 2022 data, and 0.898 and 0.617 when validated on 2021 data, respectively. This model accurately identified patients with COVID-19 at increased risk of postoperative morbidity and mortality and may provide a more objective approach to risk stratification.

## Introduction

Coronavirus disease (COVID-19), caused by severe acute respiratory syndrome coronavirus 2 (SARS-CoV-2), is known to increase the risk of perioperative morbidity and mortality both during active infection and within seven weeks of recovery [1-3]. Many patients continue to undergo surgery despite active or recent COVID-19 [4]. To improve perioperative safety, it is important to understand the risk of postoperative morbidity and mortality in vaccinated patients with COVID-19. Current guidance emphasizes infection control and the interval between COVID-19 recovery and surgery [2]. However, much of the evidence supporting these recommendations comes from studies conducted in 2020 and early 2021, before widespread vaccination and the emergence of newer SARS-CoV-2 variants [2,4-7]. Both vaccination status and variant type influence overall morbidity and mortality rates [2,3,5-8]. In the United States, single-dose vaccination coverage rose from approximately 2% at the start of 2021 to about 73% by the end of that year, and exceeded 80% in 2022 [5].

A more precise estimate of postoperative risk in a vaccinated population, including patient- and procedure-specific factors, could help optimize preoperative planning and reduce unnecessary surgical delays or cancellations. Widespread cancellations could result in losses of up to four to five billion US dollars per month in hospital system revenue [9]. Beyond the financial burden, cancellations may worsen underlying disease, negatively affect patients, further strain healthcare resources, and contribute to physician burnout [4,10].

Machine learning (ML) offers a promising tool for improving risk stratification. Early in the pandemic, several studies applied ML to predict outcomes in COVID-19 patients undergoing surgery [7,8,11]. The present study extends this work by using a large, more recent dataset, reflecting a period when a significant proportion of the US population had been vaccinated in 2021 and 2022 [5].

This investigation aimed to evaluate and predict perioperative morbidity and mortality in patients with COVID-19 who underwent major surgery in 2021 and 2022 using data from the American College of Surgeons National Surgical Quality Improvement Program (ACS NSQIP) [12,13]. The primary objective was to develop and validate ML models to predict postoperative morbidity and mortality. A secondary objective was to identify risk factors associated with poor postoperative outcomes to improve perioperative care and decision-making.

## Materials and methods

Data

The American College of Surgeons National Surgical Quality Improvement Program (ACS NSQIP) 2021 and 2022 Participant Use Data Files (PUF) were used for this investigation [12]. The 2022 dataset was used to develop predictive algorithms, and the 2021 dataset was used to validate the final models. The 2022 file included 702 participating sites and 1,011,899 surgical procedures, of which 10,613 were performed on patients with either laboratory-confirmed (10,102) or suspected (511) COVID-19 within 14 days prior to surgery. The 2021 file included 685 sites, 983,851 surgical procedures, and 5,269 patients with COVID-19 (5,032 laboratory-confirmed and 237 suspected). COVID-19 classification was based on ICD-10 diagnosis codes U07.1 or U07.2, clinical documentation of laboratory positivity, or a clinical diagnosis recorded in the medical record with or without supportive testing.

The dataset captures a wide range of perioperative risk factors, including laboratory results, comorbidities, demographics, functional status, procedure details, and anesthetic type. Postoperative complications are tracked for up to 30 days. To ensure data quality, NSQIP employs multiple safeguards, such as inter-rater reliability audits, review of operating room logs for appropriate case sampling, use of trained site-specific clinical reviewers, and an eight-day cycle schedule to ensure equal sampling probability [12].

Inclusion and exclusion criteria are specified in the PUF [12]. Generally, the dataset includes adult patients (≥18 years) undergoing major surgery at hospitals meeting quality standards. Exclusions include procedures such as hyperthermic intraperitoneal chemotherapy, organ donation, trauma, transplant, and repeat procedures within 30 days. In addition, a limited number of specific procedures are captured from each site (inguinal herniorrhaphy, breast lumpectomy, laparoscopic cholecystectomy, transurethral resection of the prostate, and transurethral resection of bladder tumor). For this investigation, all cases reported in the 2021 and 2022 PUF were included without additional inclusion or exclusion criteria.

This study did not meet the regulatory definition of human subjects research, as the data were not collected specifically for this study and were fully anonymized. The University of Pittsburgh Institutional Review Board (IRB) confirmed that review was not required. As noted by the American College of Surgeons, “the American College of Surgeons National Surgical Quality Improvement Program and the hospitals participating in the ACS NSQIP are the source of the data used herein; they have not verified and are not responsible for the statistical validity of the data analysis or the conclusions derived by the authors.”

Outcomes

The primary outcome was a binary composite of postoperative morbidity and 30-day mortality. Patients were classified as positive if they experienced any predefined complication; otherwise, they were classified as negative. Complications considered within 30 days (or shorter, if specified) included: death, unplanned re-intubation, pneumonia, pulmonary embolism, prolonged postoperative ventilation (>48 hours), myocardial infarction, cardiac arrest, new renal insufficiency or renal failure, or venous thrombosis requiring therapy. These complications were selected because they are recognized both as perioperative risks and as common complications of COVID-19 [14-16].

Data processing for machine learning

The processing steps included selection of patients with COVID-19; creation of the composite outcome; preprocessing of feature data; feature selection and engineering; application and tuning of ML models using 2022 data; final model evaluation with 2021 data; and identification of the most predictive features. Most processing was conducted in Python (version 3.11.5) with the pandas (version 2.0.1), NumPy (version 1.26.2), and scikit-learn (version 1.26.2) libraries (Python Software Foundation, Fredericksburg, VA, US).

Patient identification and outcome creation were performed as described above. Preprocessing involved handling missing data, which varied by type: missing numeric values were imputed with the median, while missing categorical values were imputed with either the most frequent category or a separate “missing” category, depending on clinical context. Although NSQIP data are not missing at random, prior studies indicate this does not significantly affect outcome prediction [17]. Rarely used categorical values were grouped into an “other” category. Current Procedural Terminology (CPT) codes were consolidated into broader procedure categories, and ICD-10 diagnosis codes were grouped by the first three characters. Additional preprocessing included linear scaling of numeric variables (0-1) and one-hot encoding of categorical variables.

Feature selection was performed manually using domain knowledge, including most preoperative variables and excluding postoperative information that could bias results. Features with no predictive value (e.g., case identifiers), little variation (e.g., case year), excessive heterogeneity (e.g., free-text procedure descriptions), or high levels of missingness (e.g., additional procedures) were also excluded. Feature engineering was limited to calculating body mass index (BMI) from height and weight. Selected preoperative laboratory values were included, though their relative timing to surgery was not considered.

To address class imbalance, the Synthetic Minority Oversampling Technique (SMOTE) was applied using the imbalanced-learn (version 0.11.0) library, which has been shown to improve model generalization and performance on minority outcomes [18,19].

Machine learning

The 2022 dataset was used to train and tune multiple ML models with repeated stratified fivefold cross-validation. Algorithms included complement Naïve Bayes, logistic regression, support vector machine, and K-nearest neighbors from the scikit-learn library. Additional models included the XGBClassifier from the xgboost library (version 2.0.3), the LGBMClassifier from the lightgbm library (version 4.2.0), and the TabNetClassifier from the pytorch-tabnet library (version 4.1.0) [20-22].

Hyperparameter optimization for all non-deep learning models was performed with either scikit-learn’s GridSearchCV or the hyperopt (version 0.2.7) and hyperopt-sklearn (version 1.0.3) libraries [23,24]. The hyperopt-sklearn library applied the “HyperoptEstimator” object, which fits classifiers using Tree-Structured Parzen Estimators [23,24].

After preprocessing and tuning, model performance was evaluated on the 2022 test set using the following metrics: overall accuracy, precision (positive predictive value, PPV), recall (sensitivity), F1 score, and the area under the receiver operating characteristic (ROC) curve (AUC).

Feature importance was assessed with Shapley Additive Explanations (SHAP), a model-agnostic method grounded in game theory that quantifies each feature’s contribution to a prediction [11,25]. Beeswarm plots were generated to illustrate the distribution of SHAP values for each feature across patients.

Statistical analysis

Statistical analysis was performed using functions from the Python statsmodels library (version 0.13.5) [26]. A two-proportion z-test was applied to compare 30-day mortality and composite outcome rates between SARS-CoV-2 positive and negative groups. Categorical variables were compared with chi-squared tests, while continuous variables were analyzed with either two-sample t-tests or, when appropriate, non-parametric Mann-Whitney U or Kruskal-Wallis tests. All test assumptions were verified before use. A significance level of α = 0.05 was set a priori, unless otherwise specified. Bonferroni correction was applied to adjust for multiple comparisons.

## Results

In the 2022 dataset, patients with COVID-19 had a significantly higher 30-day mortality rate, with 548 of 10,613 (5.2%) deaths compared to 8,858 of 1,001,286 (0.9%) among those without COVID-19 (two-tailed p < 0.001). The composite outcome, defined as 30-day mortality or selected morbidities, occurred in 1,248 of 10,613 (11.8%) patients with COVID-19, significantly higher than 29,874 of 1,001,286 (3.0%) in patients without COVID-19 (two-tailed p < 0.001).

In the 2021 dataset, 30-day mortality was also significantly higher in patients with COVID-19, with 379 of 5,269 (7.2%) deaths compared to 9,436 of 978,582 (1.0%) among those without COVID-19 (two-tailed p < 0.001). The composite outcome was present in 906 of 5,269 (17.2%) patients with COVID-19, again significantly higher than 31,019 of 978,582 (3.2%) without COVID-19 (two-tailed p < 0.001). Specific complication rates are presented in Table 1.

**Table 1:** Morbidity and mortality rates of patients with and without COVID-19 Selected morbidities and 30-day mortality rates from the 2022 and 2021 datasets are shown for patients with and without COVID-19, reported as absolute counts with corresponding frequencies (%). The composite outcome, defined as the presence of any listed morbidity or mortality, is shown in the last row.

Morbidity or mortality, n (%)	2021: COVID-19	2021: No COVID-19	2022: COVID-19	2022: No COVID-19
Death	379 (7.2%)	9436 (1.0%)	548 (5.2%)	8858 (0.9%)
Pneumonia	446 (8.5%)	10,731 (1.1%)	501 (4.7%)	10114 (1.0%)
Pulmonary embolism	54 (1.0%)	3435 (0.4%)	97 (0.9%)	3359 (0.3%)
Venous thrombosis	84 (1.6%)	5246 (0.5%)	125 (1.1%)	5248 (0.5%)
Re-intubation	154 (2.9%)	5283 (0.5%)	237 (2.2%)	4955 (0.5%)
Ventilator > 48 hours	424 (8.1%)	7324 (0.7%)	451 (4.2%)	6898 (0.7%)
Cardiac arrest	97 (1.8%)	2613 (0.3%)	126 (1.2%)	2460 (0.2%)
Myocardial infarction	59 (1.1%)	3828 (0.4%)	106 (1.0%)	3627 (0.4%)
Renal insufficiency/failure	89 (1.7%)	2181 (0.2%)	101 (1.0%)	2083 (0.2%)
Composite (any of the above)	906 (17.2%)	31,019 (3.2%)	1248 (11.8%)	29,874 (3.0%)

Comparing 2021 and 2022, patients with COVID-19 who underwent major surgery in 2021 had significantly higher mortality and composite outcome rates than those in 2022 (both two-tailed p < 0.001). Among patients without COVID-19, there were also statistically significant differences, though the absolute differences were small: 0.1% higher mortality and 0.2% higher composite outcome in 2021 compared to 2022 (p < 0.001).

All 10,613 patients with COVID-19 in the 2022 NSQIP dataset were included in the development of the ML models. Hyperparameter tuning was performed with cross-validation on an 80% randomly selected training subset of the 2022 data (8,490 procedures, 153 features). After tuning, models were evaluated on the remaining 20% test set from the 2022 data, using SMOTE oversampling. Performance results are shown in Table 2.

**Table 2:** Performance results of machine learning algorithms Performance metrics for each SMOTE-oversampled, hyperparameter-optimized, and trained machine learning model are shown in this table. Reported metrics include accuracy, precision (positive predictive value), recall (sensitivity), area under the receiver operating characteristic curve (AUC), and the F1 score. Results were obtained by training on a randomly selected subset of the 2022 data and testing on the remaining unseen 2022 test set.

	Complement Naïve Bayes	K-nearest neighbors	LightGBM	Logistic regression	Multilayer perceptron	Random forest	Support vector machine	XG boost	TabNet
Accuracy	0.794	0.726	0.858	0.812	0.808	0.904	0.835	0.879	0.912
Precision	0.337	0.259	0.433	0.359	0.352	0.644	0.385	0.490	0.720
Recall	0.780	0.716	0.672	0.756	0.748	0.412	0.680	0.612	0.336
AUC	0.876	0.802	0.884	0.880	0.874	0.876	0.863	0.880	0.862
F1 Score	0.471	0.381	0.527	0.486	0.478	0.502	0.492	0.544	0.458

**Figure 1:**
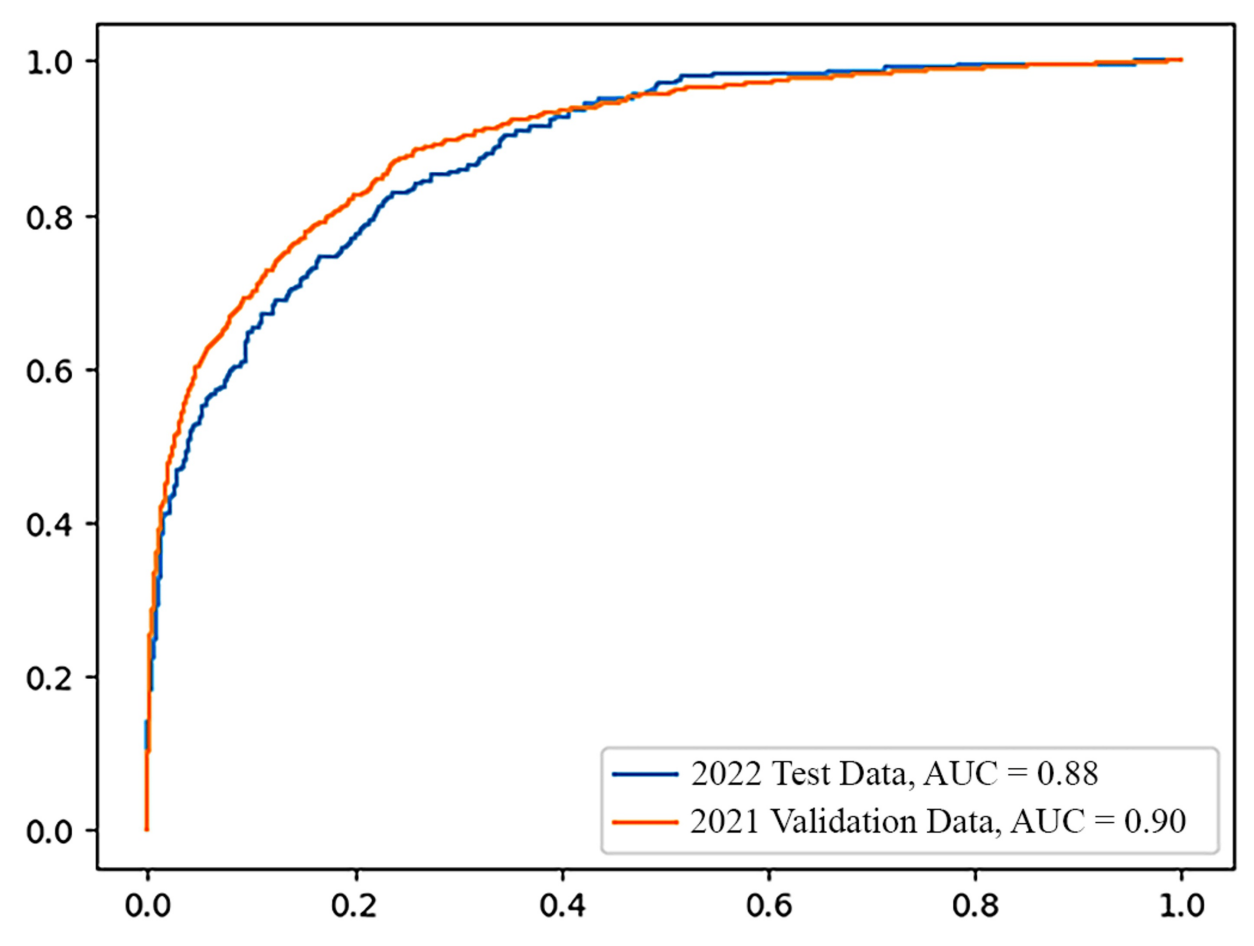
Receiver operating characteristic curve for the LightGBM model The ROC curves for the LightGBM model are shown. The blue curve represents training on a subset of the 2022 data and testing on the remaining 2022 data, with an AUC of 0.88. The orange curve represents training on the full 2022 dataset and validation on the 2021 data, with an AUC of 0.90. ROC: receiver operating characteristic, AUC: area under the receiver operating characteristic curve.

Given that the LightGBM model achieved the highest AUC and F1 scores, it was retrained on the entire 2022 dataset and then applied to the previously unseen 2021 data for validation. On the 2022 test set, the LightGBM model achieved an accuracy of 0.83, PPV of 0.38, sensitivity of 0.74, AUC of 0.88, and an F1 score of 0.50 (Table 2). On the 2021 validation set, it achieved an accuracy of 0.84, PPV of 0.52, sensitivity of 0.77, specificity of 0.85, F1 score of 0.62, and an AUC of 0.90. The ROC curves for both the 2022 test data and the 2021 validation data are shown in Figure 1.

**Figure 2:**
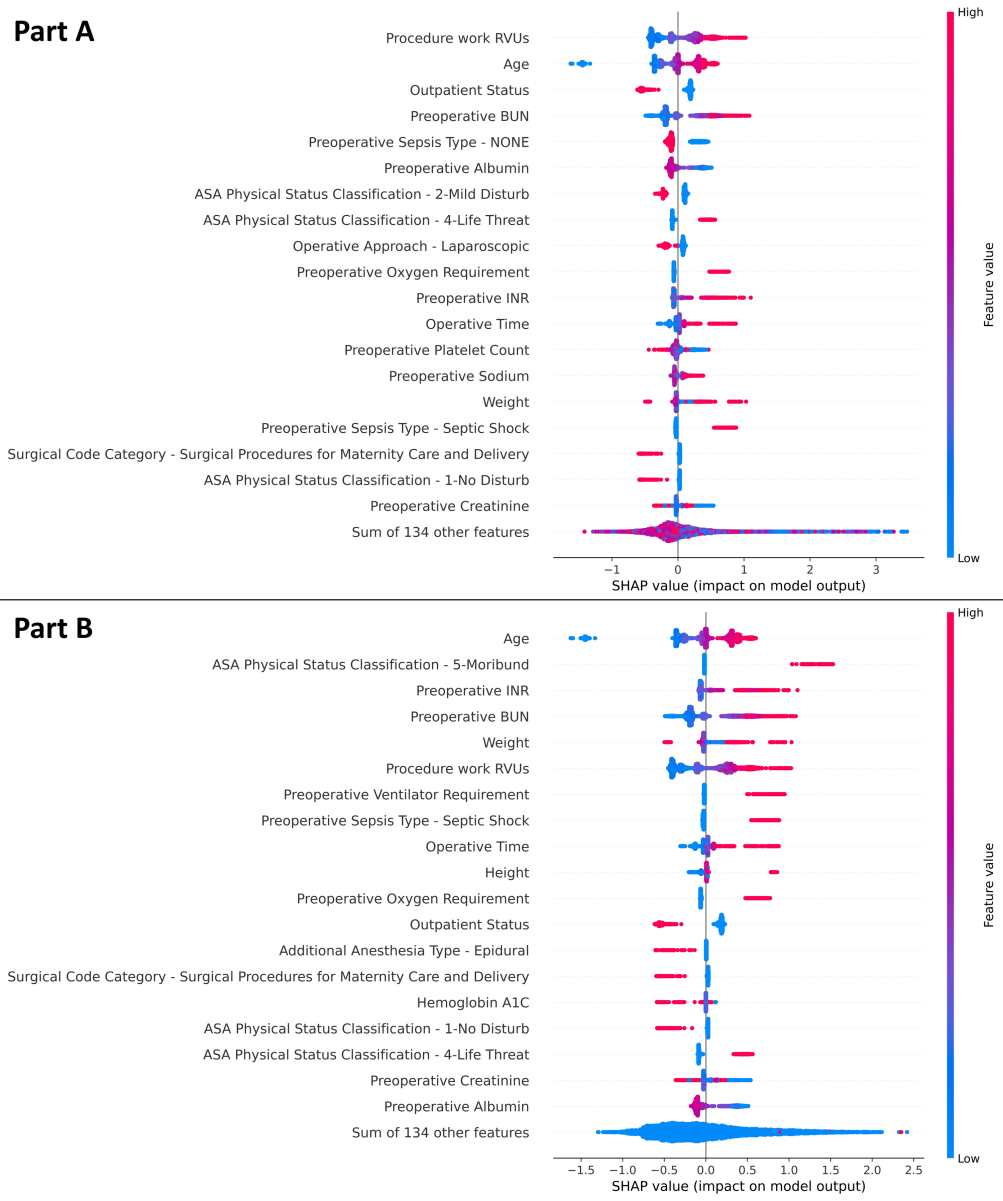
Beeswarm plots of SHAP values Beeswarm plots are shown, ordered by descending mean SHAP values (part A) and absolute maximum SHAP values (part B). The thickness of the plot at a given point reflects the density of SHAP values. Colors represent feature values, with red indicating the presence of a categorical variable or higher numeric values, and blue indicating the absence of a categorical variable or lower numeric values. The X-axis represents the SHAP value, where positive values indicate a higher likelihood of predicting the composite outcome of morbidity or mortality, and negative values indicate a higher likelihood of predicting its absence. SHAP: Shapley Additive Explanations.

SHAP values, representing feature importance, were calculated for the LightGBM model after training on the full 2022 dataset. Beeswarm plots of features with the highest mean and absolute maximum SHAP values are shown in Figure 2.

Using SHAP values, several risk factors were identified as strongly predictive of the composite outcome (Table 3). Predictors of morbidity or mortality included older age, higher work relative value units (wRVU) of the primary procedure, American Society of Anesthesiologists (ASA) Physical Status (PS) Classification IV or V, higher preoperative blood urea nitrogen (BUN), higher preoperative international normalized ratio (INR), higher weight, preoperative ventilator or oxygen requirement, preoperative septic shock, and longer operative time. Potential protective factors, predictive of absence of the composite outcome, included outpatient status, no preoperative sepsis, ASA PS II, and laparoscopic operative approach.

**Table 3:** Most predictive features for the LightGBM model The most predictive features for the LightGBM model, as determined by SHAP values, are shown in this table. Features were identified by combining those with the highest mean and maximum SHAP values. The left column lists features in descending order of importance, while the right column describes their effect on the likelihood of morbidity or mortality (composite outcome). For example, older age increases the likelihood of a positive prediction for the composite outcome.

Most predictive features (descending order of importance)	Effect of feature (i.e., model predicts (increased)/(decreased) likelihood of morbidity or mortality)
Older age	Increased
Higher work relative value units (wRVU) of primary procedure	Increased
ASA PS V classification	Increased
Outpatient status	Decreased
Higher BUN	Increased
Higher preoperative INR	Increased
No preoperative sepsis	Decreased
Higher weight	Increased
Lower serum albumin	Increased
Preoperative ventilator support	Increased
Preoperative septic shock	Increased
ASA PS II classification	Decreased
Longer operative time	Increased
ASA PS IV classification	Increased
Laparoscopic operative approach	Decreased
Preoperative oxygen support required	Increased

## Discussion

This study showed that patients with COVID-19 who underwent major surgery continued to face a higher risk of postoperative complications and mortality compared to those without COVID-19, even as vaccination rates increased throughout 2021 and 2022 [5]. Using a composite outcome of selected morbidity and 30-day mortality, we found that 906 patients (17.2%) in 2021 and 1,248 patients (11.8%) in 2022 with COVID-19 experienced the composite adverse outcome. Among patients without COVID-19, 31,019 (3.2%) in 2021 and 29,874 (3.0%) in 2022 experienced the composite outcome. These findings align with prior studies. Knisely et al. [1] reported that 16.7% of SARS-CoV-2-positive patients died after urgent or emergent surgery, compared to 1.4% of SARS-CoV-2-negative patients. Argandykov et al. [8] found higher complication and mortality rates among patients with COVID-19 (12%) compared to matched controls (8.1%). Williams et al. [27] reported an increased postoperative risk of all-cause mortality, with an odds ratio of 2.5 for patients who had COVID-19 within 45 days of ambulatory surgery in 2020 and 2021. In contrast, Garnier et al. [3] studied a more recent, largely vaccinated population during the Omicron-predominant period. Among 705 patients with recent COVID-19, 2.8% experienced a composite outcome of death or respiratory complications, a rate that was not statistically different from those without COVID-19. However, subgroup analysis revealed that patients with active symptoms on the day of surgery had an odds ratio of 4.29 for experiencing a composite respiratory outcome [3].

Several ML models accurately predicted postoperative morbidity and mortality in patients with COVID-19 who underwent major surgery. Among these, the LightGBM model was both highly performant and efficient, achieving an AUC of 0.88 and an F1 score of 0.53 on 2022 data. When validated on the 2021 dataset, it reached an AUC of 0.90 with an F1 score of 0.62. These results are consistent with prior applications of ML and logistic regression to ACS-NSQIP data outside the context of COVID-19, which reported AUC values ranging from 0.80 to 0.94 for predicting perioperative morbidity and 30-day mortality [28].

Interpretation of SHAP values from the LightGBM model highlighted several risk factors strongly associated with morbidity and mortality (Table 3). Of the 153 features evaluated, the most predictive are listed in descending order of importance. Many of these align with clinical expectations and have also been reported in prior studies using NSQIP cohorts [29].

This study has several limitations. First, as a retrospective analysis without a control group, it is subject to selection bias. For each patient, the anesthesiologist and surgeon had already determined that the benefits of proceeding with surgery outweighed the risks of delaying despite a COVID-19 diagnosis. Second, the NSQIP database has exclusion criteria that limit external validity. Third, although the overall sample size was large, COVID-19 patients comprised only a small fraction of the dataset. Fourth, because the dataset was not designed specifically for this purpose, institutional reporting and sampling of patients with COVID-19 may not reflect the broader surgical population. Finally, vaccination status and the timing of SARS-CoV-2 infection relative to surgery were not available. Future research could address these limitations by incorporating a control group, analyzing more recent data as new variants emerge, and validating findings with datasets outside of NSQIP [3,5]. Additional directions include assessing outcomes such as length of stay or applying a weighted composite outcome.

## Conclusions

This investigation analyzed a relatively large cohort of patients with COVID-19 who underwent major surgery during 2021-2022, a period of widespread and rapidly increasing SARS-CoV-2 vaccination in the United States. As expected, higher rates of major postoperative morbidity and 30-day mortality were observed in both years. Using a machine learning approach, several models accurately predicted postoperative outcomes based on preoperative data. The LightGBM model, developed on 2022 data and validated on 2021 data, demonstrated strong performance. Among the 153 features evaluated, multiple risk factors were identified as strongly predictive of morbidity and mortality. Similar to existing risk scoring systems, prospective application of this approach for surgical patients with COVID-19 could provide more objective risk stratification and enhance perioperative decision-making in the post-vaccination era.
